# Supplementary material for: Altered microRNA Transcriptome in Cultured Human Liver Cells upon Infection with Ebola Virus
Source: Int J Mol Sci. 2021 Apr 6;22(7):3792. doi: 10.3390/ijms22073792 (PMC8038836; doi:10.3390/ijms22073792)
Supplement: Supplementary file 1 [file ijms-22-03792-s001.zip › Supplementary_File/C_ GO_Analysis_Results/16-30nt_go_Mayinga-24h-Huh7_vs_Control-24h-Huh7_down.mature_mirna_targets/MF_result(Human).html]

| GO.ID | Term | Ontology | Count | Pop.Hits | List.Total | Pop.Total | Fold.Enrichment | Pvalue | FDR | Enrichment.Score | Gene.Ratio | GENES |
| --- | --- | --- | --- | --- | --- | --- | --- | --- | --- | --- | --- | --- |
| GO:0005515 | protein binding | Molecular function | 136 | 11565 | 172 | 17548 | 1.19975467277974 | 0.000102337959710238 | 0.132629995784468 | 3.98996324571272 | 0.790697674418605 | CPLX1//GOSR2//EP300//LMO2//SRF//FAM89B//FOXO1//GNAO1//GNAZ//NUCB1//MIF//ZNF385B//SPTBN5//TWF1//MLPH//MICAL2//TOB1//ARHGEF6//GRIN2B//NCAM1//CAMK2A//ABCA1//NDP//SFRP1//BDNF//NPNT//ADAM11//FERMT3//CRIM1//PTPRT//KIF2A//FAM161A//VAPA//PRKAA1//YWHAB//DYNLL1//CSPG5//CDH7//HEYL//APBB1//PURA//HDAC11//SH3BP4//DENND6A//ST5//SH3BP5//GPC1//FGFRL1//RIMS4//TRABD2B//RALBP1//UBE4B//TTC28//CDKN2D//OSR1//RHOF//CARHSP1//PPP1R26//ARFIP2//SEMA4B//SEMA4G//RAB3B//FAF2//ISCU//FOXP3//ASF1B//HAT1//NUDT16//P2RX2//SULT4A1//MCIDAS//CNTN2//BEST1//GPD1L//LHPP//CREB3L3//UBXN2B//TP53INP2//SRSF3//PFDN1//KCNIP3//SCN3B//EFNA3//ANO3//BRK1//AKAP10//SHQ1//DPYS//PYURF//ENAM//TOMM40//LYPD6//PACRGL//USP54//BAHD1//MGRN1//ATP1B4//CNIH2//ARMC8//NAT9//MYCBP//PTPN18//GPR3//ZDHHC22//HOXC9//ARL16//IL4R//FAM111B//MEA1//NDUFA4//ARL6IP4//ARID4B//POLR2E//FAM86C1//VPS53//THUMPD1//PRKCQ//PSMB8//ATXN7L3//PCTP//GPATCH3//COPS7B//SMPD1//BTG2//PAX8//CCDC134//NUP214//KMT2D//FAM167A//CASP7//OGT//CGNL1//CYP4F2//SESTD1//FAM131B//MFAP3L// |
| GO:0005488 | binding | Molecular function | 161 | 15006 | 172 | 17548 | 1.09461176769602 | 0.000729856121427075 | 0.472946766684745 | 3.13676274512457 | 0.936046511627907 | CPLX1//GOSR2//NUDT16//LHPP//PURA//BAHD1//SRF//CREB3L3//ARHGAP35//SP5//LMO2//EP300//KCNIP3//ZNF395//PAX8//HEYL//FOXP3//FAM89B//FOXO1//APBB1//GNAO1//GNAZ//NUCB1//MIF//ZNF385B//DPYS//OSR1//SPRN//GPATCH3//ZNF618//POLR3H//CARHSP1//POLR2E//KMT2D//BTAF1//PRKAA1//RPP14//ARL6IP4//THUMPD1//SRSF3//NOL4//MEX3A//SPTBN5//TWF1//MLPH//MICAL2//TOB1//ARHGEF6//GRIN2B//NCAM1//CAMK2A//ABCA1//NDP//SFRP1//BDNF//NPNT//ADAM11//FERMT3//ISCU//CYP4F3//CYP4F2//GPC1//CDH7//MYL10//PYURF//ENAM//CDKN2D//TOMM40//CSPG5//RALBP1//PTPRT//AKAP10//LYPD6//PACRGL//UBXN2B//USP54//EFNA3//FAF2//MGRN1//ATP1B4//ARFIP2//SH3BP4//CNIH2//SULT4A1//ARMC8//NAT9//MYCBP//PTPN18//GPR3//ZDHHC22//HOXC9//ARL16//MCIDAS//IL4R//FAM111B//KIF2A//TRABD2B//MEA1//NDUFA4//ARID4B//SHQ1//FAM86C1//VPS53//ASF1B//BRK1//PRKCQ//PSMB8//ATXN7L3//SEMA4G//TP53INP2//PCTP//RAB3B//COPS7B//SMPD1//YWHAB//BTG2//CCDC134//NUP214//FAM167A//CASP7//FAM161A//OGT//CGNL1//HAT1//DYNLL1//SESTD1//VAPA//SH3BP5//FAM131B//MFAP3L//PPP1R26//CRIM1//UBE4B//STK35//P2RX2//RABL3//RHOF//PLA2G15//HDAC11//CHRM1//FGFRL1//ZSWIM6//DDAH1//DENND6A//ST5//RIMS4//TTC28//SEMA4B//CLEC1A//CNTN2//TULP4//BEST1//GPD1L//PFDN1//SCN3B//PRUNE2//RNF215//NPTX1//BSN//B4GALT5//ANO3// |
| GO:0031267 | small GTPase binding | Molecular function | 14 | 570 | 172 | 17548 | 2.50583435332517 | 0.00149119045564877 | 0.636373241729905 | 2.82646688467916 | 0.0813953488372093 | GRIN2B//NCAM1//SPTBN5//CAMK2A//ARHGEF6//SH3BP4//DENND6A//ST5//RIMS4//MLPH//RALBP1//ARFIP2//BRK1//ABCA1// |
| GO:0017016 | Ras GTPase binding | Molecular function | 13 | 527 | 172 | 17548 | 2.51670270508804 | 0.00211043005138649 | 0.636373241729905 | 2.67562903763433 | 0.0755813953488372 | GRIN2B//NCAM1//SPTBN5//CAMK2A//ARHGEF6//DENND6A//ST5//RIMS4//MLPH//RALBP1//ARFIP2//BRK1//SH3BP4// |
| GO:0016407 | acetyltransferase activity | Molecular function | 5 | 105 | 172 | 17548 | 4.85825027685493 | 0.00374556168463291 | 0.636373241729905 | 2.42648304625681 | 0.0290697674418605 | EP300//HAT1//NAT9//OGT//MOGAT2// |
| GO:0051020 | GTPase binding | Molecular function | 14 | 665 | 172 | 17548 | 2.14785801713586 | 0.00592594346836028 | 0.636373241729905 | 2.22724249586918 | 0.0813953488372093 | ARHGEF6//GRIN2B//NCAM1//SPTBN5//CAMK2A//SH3BP4//DENND6A//ST5//RIMS4//MLPH//RALBP1//ABCA1//ARFIP2//BRK1// |
| GO:0016747 | transferase activity, transferring acyl groups other than amino-acyl groups | Molecular function | 7 | 221 | 172 | 17548 | 3.23150584026097 | 0.00620139806657627 | 0.636373241729905 | 2.20751039047713 | 0.0406976744186047 | MOGAT2//EP300//HAT1//NAT9//PLA2G15//ZDHHC22//OGT// |
| GO:0016709 | oxidoreductase activity, acting on paired donors, with incorporation or reduction of molecular oxygen, NAD(P)H as one donor, and incorporation of one atom of oxygen | Molecular function | 3 | 44 | 172 | 17548 | 6.9561310782241 | 0.00912956981538189 | 0.636373241729905 | 2.03954968590572 | 0.0174418604651163 | CYP4F3//CYP4F2//MICAL2// |
| GO:0001056 | RNA polymerase III activity | Molecular function | 2 | 15 | 172 | 17548 | 13.6031007751938 | 0.00922291109222905 | 0.636373241729905 | 2.03513197788943 | 0.0116279069767442 | POLR3H//POLR2E// |
| GO:0008080 | N-acetyltransferase activity | Molecular function | 4 | 86 | 172 | 17548 | 4.74526771227691 | 0.010151572557403 | 0.636373241729905 | 1.99346667695249 | 0.0232558139534884 | EP300//HAT1//OGT//NAT9// |
| GO:0000989 | transcription factor activity, transcription factor binding | Molecular function | 13 | 643 | 172 | 17548 | 2.06267857788708 | 0.0108612482603068 | 0.636373241729905 | 1.96412025933188 | 0.0755813953488372 | SRF//HEYL//LMO2//EP300//MYCBP//MCIDAS//ATXN7L3//TOB1//ARHGAP35//KCNIP3//FOXP3//YWHAB//FOXO1// |
| GO:0005525 | GTP binding | Molecular function | 9 | 371 | 172 | 17548 | 2.47495768820911 | 0.0110665991761568 | 0.636373241729905 | 1.95598581958599 | 0.0523255813953488 | NUDT16//ARFIP2//GNAO1//GNAZ//RABL3//ARHGAP35//ARL16//RHOF//RAB3B// |
| GO:0000988 | transcription factor activity, protein binding | Molecular function | 13 | 648 | 172 | 17548 | 2.04676284811944 | 0.0115296341463514 | 0.636373241729905 | 1.93818447334204 | 0.0755813953488372 | FOXO1//HEYL//SRF//LMO2//EP300//MYCBP//MCIDAS//ATXN7L3//TOB1//ARHGAP35//KCNIP3//FOXP3//YWHAB// |
| GO:0032550 | purine ribonucleoside binding | Molecular function | 9 | 374 | 172 | 17548 | 2.45510508643204 | 0.011618194888033 | 0.636373241729905 | 1.93486134277738 | 0.0523255813953488 | NUDT16//ARFIP2//GNAO1//GNAZ//RABL3//ARHGAP35//ARL16//RHOF//RAB3B// |
| GO:0005088 | Ras guanyl-nucleotide exchange factor activity | Molecular function | 7 | 251 | 172 | 17548 | 2.84527008246085 | 0.0120090758117807 | 0.636373241729905 | 1.9204904135203 | 0.0406976744186047 | ARHGEF6//DENND6A//ST5//GRIN2B//NCAM1//SPTBN5//CAMK2A// |
| GO:0001883 | purine nucleoside binding | Molecular function | 9 | 377 | 172 | 17548 | 2.43556844118191 | 0.0121901569784282 | 0.636373241729905 | 1.91399070172976 | 0.0523255813953488 | NUDT16//ARFIP2//GNAO1//GNAZ//RABL3//ARHGAP35//ARL16//RHOF//RAB3B// |
| GO:0001102 | RNA polymerase II activating transcription factor binding | Molecular function | 3 | 49 | 172 | 17548 | 6.24632178452776 | 0.0122569452174871 | 0.636373241729905 | 1.91161775498056 | 0.0174418604651163 | EP300//LMO2//SRF// |
| GO:0032549 | ribonucleoside binding | Molecular function | 9 | 378 | 172 | 17548 | 2.42912513842746 | 0.0123854128917093 | 0.636373241729905 | 1.90708951078973 | 0.0523255813953488 | NUDT16//ARFIP2//GNAO1//GNAZ//RABL3//ARHGAP35//ARL16//RHOF//RAB3B// |
| GO:0016746 | transferase activity, transferring acyl groups | Molecular function | 7 | 253 | 172 | 17548 | 2.82277782884456 | 0.0125010828670182 | 0.636373241729905 | 1.90305236596781 | 0.0406976744186047 | MOGAT2//EP300//HAT1//NAT9//PLA2G15//ZDHHC22//OGT// |
| GO:0098772 | molecular function regulator | Molecular function | 28 | 1848 | 172 | 17548 | 1.54580690627202 | 0.0129761893357314 | 0.636373241729905 | 1.88685282629079 | 0.162790697674419 | ATP1B4//SH3BP5//CDKN2D//PPP1R26//CRIM1//ARHGEF6//GRIN2B//NCAM1//SPTBN5//CAMK2A//SH3BP4//RALBP1//ARHGAP35//MIF//NDP//CSPG5//BDNF//KCNIP3//GPD1L//SCN3B//DENND6A//ST5//UBXN2B//DYNLL1//LYPD6//SEMA4B//SEMA4G//FAF2// |
| GO:0010485 | H4 histone acetyltransferase activity | Molecular function | 2 | 18 | 172 | 17548 | 11.3359173126615 | 0.0131831592446663 | 0.636373241729905 | 1.87998050187307 | 0.0116279069767442 | OGT//HAT1// |
| GO:0001882 | nucleoside binding | Molecular function | 9 | 385 | 172 | 17548 | 2.38495922681969 | 0.0138182977575367 | 0.636373241729905 | 1.85954545334688 | 0.0523255813953488 | NUDT16//ARFIP2//GNAO1//GNAZ//RABL3//ARHGAP35//ARL16//RHOF//RAB3B// |
| GO:0016462 | pyrophosphatase activity | Molecular function | 16 | 892 | 172 | 17548 | 1.83001355720096 | 0.0144616283036333 | 0.636373241729905 | 1.83978280500346 | 0.0930232558139535 | KIF2A//CGNL1//GNAO1//GNAZ//RABL3//ARHGAP35//RHOF//RAB3B//PRUNE2//BTAF1//LHPP//ATP1B4//DYNLL1//RALBP1//ABCA1//NUDT16// |
| GO:0005229 | intracellular calcium activated chloride channel activity | Molecular function | 2 | 19 | 172 | 17548 | 10.7392900856793 | 0.0146401346306905 | 0.636373241729905 | 1.83445492948684 | 0.0116279069767442 | ANO3//BEST1// |
| GO:0038191 | neuropilin binding | Molecular function | 2 | 19 | 172 | 17548 | 10.7392900856793 | 0.0146401346306905 | 0.636373241729905 | 1.83445492948684 | 0.0116279069767442 | SEMA4B//SEMA4G// |
| GO:0061778 | intracellular chloride channel activity | Molecular function | 2 | 19 | 172 | 17548 | 10.7392900856793 | 0.0146401346306905 | 0.636373241729905 | 1.83445492948684 | 0.0116279069767442 | ANO3//BEST1// |
| GO:0016817 | hydrolase activity, acting on acid anhydrides | Molecular function | 16 | 894 | 172 | 17548 | 1.82591956714011 | 0.0147427417422716 | 0.636373241729905 | 1.83142174220209 | 0.0930232558139535 | KIF2A//CGNL1//GNAO1//GNAZ//RABL3//ARHGAP35//RHOF//RAB3B//PRUNE2//BTAF1//LHPP//ATP1B4//DYNLL1//RALBP1//ABCA1//NUDT16// |
| GO:0016818 | hydrolase activity, acting on acid anhydrides, in phosphorus-containing anhydrides | Molecular function | 16 | 894 | 172 | 17548 | 1.82591956714011 | 0.0147427417422716 | 0.636373241729905 | 1.83142174220209 | 0.0930232558139535 | KIF2A//CGNL1//GNAO1//GNAZ//RABL3//ARHGAP35//RHOF//RAB3B//PRUNE2//BTAF1//LHPP//ATP1B4//DYNLL1//RALBP1//ABCA1//NUDT16// |
| GO:0032561 | guanyl ribonucleotide binding | Molecular function | 9 | 391 | 172 | 17548 | 2.34836138702195 | 0.0151414831564597 | 0.636373241729905 | 1.81983158222577 | 0.0523255813953488 | NUDT16//ARFIP2//GNAO1//GNAZ//RABL3//ARHGAP35//ARL16//RHOF//RAB3B// |
| GO:0019001 | guanyl nucleotide binding | Molecular function | 9 | 392 | 172 | 17548 | 2.34237066919791 | 0.0153708018185459 | 0.636373241729905 | 1.81330347691878 | 0.0523255813953488 | NUDT16//ARFIP2//GNAO1//GNAZ//RABL3//ARHGAP35//ARL16//RHOF//RAB3B// |
| GO:0030215 | semaphorin receptor binding | Molecular function | 2 | 20 | 172 | 17548 | 10.2023255813953 | 0.016163138030751 | 0.636373241729905 | 1.79147431824363 | 0.0116279069767442 | SEMA4B//SEMA4G// |
| GO:0031683 | G-protein beta/gamma-subunit complex binding | Molecular function | 2 | 20 | 172 | 17548 | 10.2023255813953 | 0.016163138030751 | 0.636373241729905 | 1.79147431824363 | 0.0116279069767442 | GNAO1//GNAZ// |
| GO:0005548 | phospholipid transporter activity | Molecular function | 3 | 56 | 172 | 17548 | 5.46553156146179 | 0.0175488782987082 | 0.636373241729905 | 1.75575063784488 | 0.0174418604651163 | PCTP//ANO3//ABCA1// |
| GO:0045499 | chemorepellent activity | Molecular function | 2 | 21 | 172 | 17548 | 9.71650055370986 | 0.0177507508138019 | 0.636373241729905 | 1.75078327261481 | 0.0116279069767442 | SEMA4B//SEMA4G// |
| GO:0004402 | histone acetyltransferase activity | Molecular function | 3 | 57 | 172 | 17548 | 5.36964504283966 | 0.0183932775938702 | 0.636373241729905 | 1.73534087467171 | 0.0174418604651163 | HAT1//OGT//EP300// |
| GO:0016410 | N-acyltransferase activity | Molecular function | 4 | 103 | 172 | 17548 | 3.96206818694965 | 0.0186118837233015 | 0.636373241729905 | 1.73020966936068 | 0.0232558139534884 | EP300//HAT1//NAT9//OGT// |
| GO:0005543 | phospholipid binding | Molecular function | 9 | 410 | 172 | 17548 | 2.23953488372093 | 0.0199503757924971 | 0.636373241729905 | 1.70004891937218 | 0.0523255813953488 | TWF1//SESTD1//OGT//PCTP//TULP4//ARFIP2//ABCA1//PLA2G15//ARHGAP35// |
| GO:0061733 | peptide-lysine-N-acetyltransferase activity | Molecular function | 3 | 59 | 172 | 17548 | 5.18762317698069 | 0.0201488548669819 | 0.636373241729905 | 1.69574963136322 | 0.0174418604651163 | EP300//HAT1//OGT// |
| GO:0016597 | amino acid binding | Molecular function | 3 | 60 | 172 | 17548 | 5.10116279069767 | 0.0210600819254801 | 0.636373241729905 | 1.67653994370539 | 0.0174418604651163 | GRIN2B//DPYS//DDAH1// |
| GO:0000983 | transcription factor activity, RNA polymerase II core promoter sequence-specific DNA binding | Molecular function | 2 | 23 | 172 | 17548 | 8.87158746208291 | 0.0211142356438163 | 0.636373241729905 | 1.67542463584368 | 0.0116279069767442 | HEYL//SRF// |
| GO:0015106 | bicarbonate transmembrane transporter activity | Molecular function | 2 | 23 | 172 | 17548 | 8.87158746208291 | 0.0211142356438163 | 0.636373241729905 | 1.67542463584368 | 0.0116279069767442 | SLC26A1//BEST1// |
| GO:0017134 | fibroblast growth factor binding | Molecular function | 2 | 23 | 172 | 17548 | 8.87158746208291 | 0.0211142356438163 | 0.636373241729905 | 1.67542463584368 | 0.0116279069767442 | GPC1//FGFRL1// |
| GO:0030742 | GTP-dependent protein binding | Molecular function | 2 | 23 | 172 | 17548 | 8.87158746208291 | 0.0211142356438163 | 0.636373241729905 | 1.67542463584368 | 0.0116279069767442 | ARFIP2//RAB3B// |
| GO:0017022 | myosin binding | Molecular function | 3 | 63 | 172 | 17548 | 4.85825027685493 | 0.0239276501444894 | 0.704778058801324 | 1.62109994991942 | 0.0174418604651163 | RAB3B//SPTBN5//MLPH// |
| GO:0070273 | phosphatidylinositol-4-phosphate binding | Molecular function | 2 | 25 | 172 | 17548 | 8.16186046511628 | 0.0247196588021798 | 0.711926173502778 | 1.60695752797453 | 0.0116279069767442 | ARFIP2//SESTD1// |
| GO:0044212 | transcription regulatory region DNA binding | Molecular function | 15 | 877 | 172 | 17548 | 1.74498157036408 | 0.0257338415951294 | 0.713135836861494 | 1.58949537667226 | 0.0872093023255814 | BAHD1//SRF//CREB3L3//ARHGAP35//SP5//LMO2//PURA//EP300//KCNIP3//ZNF395//PAX8//HEYL//FOXP3//ARID4B//KMT2D// |
| GO:0001067 | regulatory region nucleic acid binding | Molecular function | 15 | 878 | 172 | 17548 | 1.74299411982836 | 0.0259619594818928 | 0.713135836861494 | 1.58566253220841 | 0.0872093023255814 | BAHD1//SRF//CREB3L3//ARHGAP35//SP5//LMO2//PURA//EP300//KCNIP3//ZNF395//PAX8//HEYL//FOXP3//ARID4B//KMT2D// |
| GO:0003714 | transcription corepressor activity | Molecular function | 6 | 232 | 172 | 17548 | 2.63853247794707 | 0.0269720951607149 | 0.713135836861494 | 1.56908531675083 | 0.0348837209302326 | HEYL//TOB1//ARHGAP35//KCNIP3//FOXP3//YWHAB// |
| GO:0004714 | transmembrane receptor protein tyrosine kinase activity | Molecular function | 3 | 66 | 172 | 17548 | 4.63742071881607 | 0.0269959379985613 | 0.713135836861494 | 1.56870157797151 | 0.0174418604651163 | EFNA3//FGFRL1//CRIM1// |
| GO:0034212 | peptide N-acetyltransferase activity | Molecular function | 3 | 67 | 172 | 17548 | 4.56820548420687 | 0.0280632158024199 | 0.713135836861494 | 1.55186256405522 | 0.0174418604651163 | EP300//HAT1//OGT// |
| GO:0048365 | Rac GTPase binding | Molecular function | 3 | 67 | 172 | 17548 | 4.56820548420687 | 0.0280632158024199 | 0.713135836861494 | 1.55186256405522 | 0.0174418604651163 | RALBP1//ARFIP2//BRK1// |
| GO:0043130 | ubiquitin binding | Molecular function | 3 | 68 | 172 | 17548 | 4.50102599179207 | 0.0291526973879716 | 0.725693182101657 | 1.53532125543277 | 0.0174418604651163 | UBXN2B//FAF2//TP53INP2// |
| GO:0044325 | ion channel binding | Molecular function | 4 | 119 | 172 | 17548 | 3.42935313660348 | 0.0296772674779227 | 0.725693182101657 | 1.52757608903626 | 0.0232558139534884 | RIMS4//GPD1L//KCNIP3//SCN3B// |
| GO:0045505 | dynein intermediate chain binding | Molecular function | 2 | 28 | 172 | 17548 | 7.28737541528239 | 0.0305583177971486 | 0.733399627131566 | 1.51487055688706 | 0.0116279069767442 | SPTBN5//DYNLL1// |
| GO:0033613 | activating transcription factor binding | Molecular function | 3 | 73 | 172 | 17548 | 4.19273654029946 | 0.0349314948467227 | 0.818994515438183 | 1.45678282891168 | 0.0174418604651163 | EP300//LMO2//SRF// |
| GO:0016787 | hydrolase activity | Molecular function | 35 | 2636 | 172 | 17548 | 1.35463351801532 | 0.0353886519016499 | 0.818994515438183 | 1.45113598109873 | 0.203488372093023 | KIF2A//CGNL1//GNAO1//GNAZ//RABL3//ARHGAP35//RHOF//RAB3B//DPYS//PSMB8//SFRP1//CASP7//TRABD2B//ADAM11//PRUNE2//BTAF1//ARID4B//HDAC11//LHPP//CHRM1//RPP14//PLA2G15//CTDSPL2//PTPRT//PTPN18//SMPD1//ATP1B4//NUDT16//ABHD17B//DYNLL1//DDAH1//RALBP1//ABCA1//USP54//TMEM62// |
| GO:0017147 | Wnt-protein binding | Molecular function | 2 | 31 | 172 | 17548 | 6.5821455363841 | 0.0368830932992412 | 0.838605068698537 | 1.43317266282297 | 0.0116279069767442 | TRABD2B//SFRP1// |
| GO:0005085 | guanyl-nucleotide exchange factor activity | Molecular function | 7 | 323 | 172 | 17548 | 2.21103031175751 | 0.0402248880129919 | 0.898818187324785 | 1.39550515631302 | 0.0406976744186047 | GRIN2B//NCAM1//SPTBN5//CAMK2A//ARHGEF6//DENND6A//ST5// |
| GO:0000981 | RNA polymerase II transcription factor activity, sequence-specific DNA binding | Molecular function | 17 | 1108 | 172 | 17548 | 1.56533876248846 | 0.0444839583276438 | 0.977139152417396 | 1.35179657476758 | 0.0988372093023256 | SRF//HEYL//PAX8//FOXO1//KCNIP3//BTG2//ARHGAP35//PURA//EP300//LMO2//CREB3L3//FOXP3//CARHSP1//HOXC9//SP5//ZNF395//KMT2D// |
| GO:0005254 | chloride channel activity | Molecular function | 3 | 82 | 172 | 17548 | 3.73255813953488 | 0.0467032005627241 | 0.985821882697762 | 1.33065335628397 | 0.0174418604651163 | ANO3//BEST1//SLC26A1// |
| GO:0008013 | beta-catenin binding | Molecular function | 3 | 82 | 172 | 17548 | 3.73255813953488 | 0.0467032005627241 | 0.985821882697762 | 1.33065335628397 | 0.0174418604651163 | PTPRT//EP300//FOXO1// |
| GO:0019199 | transmembrane receptor protein kinase activity | Molecular function | 3 | 83 | 172 | 17548 | 3.68758755954049 | 0.048117643612825 | 0.985821882697762 | 1.31769564880943 | 0.0174418604651163 | EFNA3//FGFRL1//CRIM1// |
| GO:0000979 | RNA polymerase II core promoter sequence-specific DNA binding | Molecular function | 2 | 36 | 172 | 17548 | 5.66795865633075 | 0.0484159229558781 | 0.985821882697762 | 1.31501178474794 | 0.0116279069767442 | HEYL//PAX8// |
